# Health risk assessment upon exposure to groundwater arsenic among individuals of different sex and age groups of Vaishali district, Bihar (India)

**DOI:** 10.1016/j.toxrep.2025.102024

**Published:** 2025-04-09

**Authors:** Pankaj Kumar, Parimal Kumar Khan, Amod Kumar

**Affiliations:** a Department of Zoology, Patna University, Patna 800005, India; b Department of Zoology, University of Delhi, New Delhi 110007, India

**Keywords:** Cancer index, Cancer risk, Contamination factor, Ganga River Basin, Hazard index, Non-cancer risk

## Abstract

Availability of safe drinking water is one of the requirements for maintaining good health. Unfortunately, inhabitants of many nations suffer from adverse health effects due to the intake of arsenic-contaminated groundwater. The Vaishali district of Bihar (India) is the part of Ganga River Basin, a hotspot of arsenic contamination and hence, risk assessment among its individuals is highly pertinent. This study aimed to evaluate the extent of arsenic contamination in the ground waters of Bidupur block under Vaishali district, followed by an assessment of health risk, both non-cancer and cancer, within the arsenic-exposed adult females, adult males and children. Estimation of groundwater arsenic was done in 68 duplicate samples through an MQuant test kit (Merck, Germany). For this, Microsoft Office Excel and ArcGIS software were used as a tool. The results showed that only one-fourth of the groundwater samples exceeded the WHO permissible limit of arsenic with a high contamination factor. The total hazard index (HI), representing the non-cancer risk, was found above the threshold value (>1) among all individuals, which was high among the adults, more in adult females (3.21) than adult males (2.97), and low among the children (2.02). The cancer risk, expressed in terms of cancer index (CI), was also beyond the acceptable limit (10^−4^ to 10^−6^) among all sex and age groups, ranging from 0.91 × 10^−3^ to 1.45 × 10^−3^. Conclusively, arsenic was found to pose both high non-cancer and cancer risks in the population even at its low level due to long-term exposure.

## Highlights

• Health risk of individuals exposed to groundwater arsenic was assessed in Vaishali district, of Bihar (India).
• Only one-fourth of groundwater samples contained arsenic beyond its permissible limit.
• Non-cancer risk as well as cancer risk were above the acceptable limit among all individuals.
• Risk was high among adult females than adult males and low among children.
• Arsenic was posing high health risk even at its low level due to long-term exposure.

## Graphical Abstract

**Figure:**
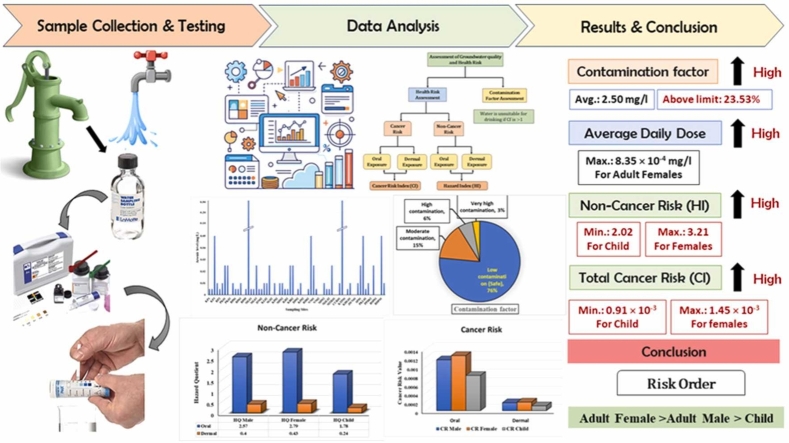

## 1 Introduction

Groundwater is a precious natural resource for drinking water [1]. As around 2.4 billion people live in water-stressed countries and another 2.2 billion people lack safe water management, increasing demand for clean and accessible water may take the form of a silent revolution [2], [3]. However, in recent years, both human activities and natural factors have immensely degraded the quality of groundwater, especially in alluvial plains [4], [5], [6], [7], [8]. Contamination of heavy metals, like arsenic, fluoride and iron, are frequently reported in the groundwater of alluvial plains of the Ganga River Basin which poses serious environmental challenges, and becomes a global issue due to their widespread presence, environmental persistence, and toxicity [9], [10], [11], [12], [13]. Heavy metals are resistant to microbial and chemical degradation, and can have harmful physiological effects on living organisms, even their trace amounts are considered harmful [14], [15]. These metals have the ability to build up in living organisms, such as plants and animals, ultimately making their way into the human food chain and possibly leading to health issues [16]. The rapid industrial expansion and the heightened release of agrochemicals into the environment have amplified toxic consequences of heavy metals in human systems [17], [18].

Arsenic is a highly toxic metalloid, and a group I human carcinogen [19], [20] which has been studied extensively for their adverse health effects [21], [22], [23], [24]. Its trivalent form (arsenite) is found more toxic than pentavalent form (arsenate) because the former reacts more frequently, and has less excretion rate (60–75 %) from the human body [25], [26]. Its accumulation and occurrence above the WHO permissible limit put over 230 million people in danger of arsenic poisoning globally, including 180 million in Asia alone [2]. Groundwater arsenic contamination occurs due to geogenic (weathering of rocks, geothermal, and volcanic activities in deep aquifers) as well as anthropogenic activities (mining processes, smelting, pesticide application, and waste incineration). Several aquifers have been identified worldwide as having arsenic levels higher than its WHO permissible limit [2], [5], [27], [28], [29], [30], and are mainly found in Bangladesh [31], China [32], Pakistan [33], India [2], Nepal [34], Argentina [35], and several places in the United States [36]. In India, 20 states and 4 union territories have areas where groundwater arsenic levels are beyond the WHO permissible limit, out of which 5 states, including Bihar along the Ganga River Basin, are severely affected [12], [37], [38].

Arsenic enters the human body mainly through the consumption of groundwater and agro-products grown using arsenic-contaminated groundwater [21], [39], [40], [41], [42]. Prolonged exposure to arsenic causes several adverse health effects which include keratosis, melanosis and anomalies in several systems, such as cardiovascular, gastrointestinal, endocrine and reproductive [43], [44] as well as numerous types of cancers [45]. Moreover, even low levels of arsenic exposure can also cause genotoxic effects such as micronucleus induction and DNA fragmentation [46], [47].

In recent years, researchers have started to focus on assessing the human health risk caused by heavy metals besides their spatial variation [41], [48], [49]. Several health risk indices such as hazard index (HI), hazard quotient (HQ), and cancer risk (CR) have been developed and applied to evaluate the potential hazards associated with freshwater contaminants [40], [41], [50], [51], [52], [53], [54]. Such studies, performed in India and abroad, are based on exposure assessment and hazard identification to estimate non-cancer and cancer risks in the population using the guidelines of the United States Environmental Protection Agency (USEPA) [40], [41], [54], [55], [56].

According to the Economic Survey Report of Bihar (2021–2022), 31 out of 38 districts are currently suffering from increased levels of arsenic [57]. The Vaishali district of Bihar (India), situated along the Ganga River Basin, is considered one of the high-risk zones of arsenic contamination in groundwater [6], [7]. However, no study has been carried out for the human health risk assessment in this area. Hence, this study aimed to (1) investigate the level of groundwater arsenic in the area, (2) explore its contamination factor, and (3) assess the human health risk caused by groundwater arsenic. This study will make a substantial contribution in assessing the health risk of groundwater arsenic exposure in the human population of the Vaishali district, Bihar (India).

## 2 Materials and methods

### 2.1 Study area

The study area, Bidupur block of Vaishali district, is located in Ganga River Basin on the northern bank of river Ganga at 25°64’97’’ N latitude and 85°32’69’’ E longitude within the state of Bihar, India, with a land area of 2036 km^2^. This block has a total of 116 villages inhabited by a population of 268,849 people (143,090 males and 125,759 females) with a poor sex ratio of 879/1000 [58].

**Fig. 1:**
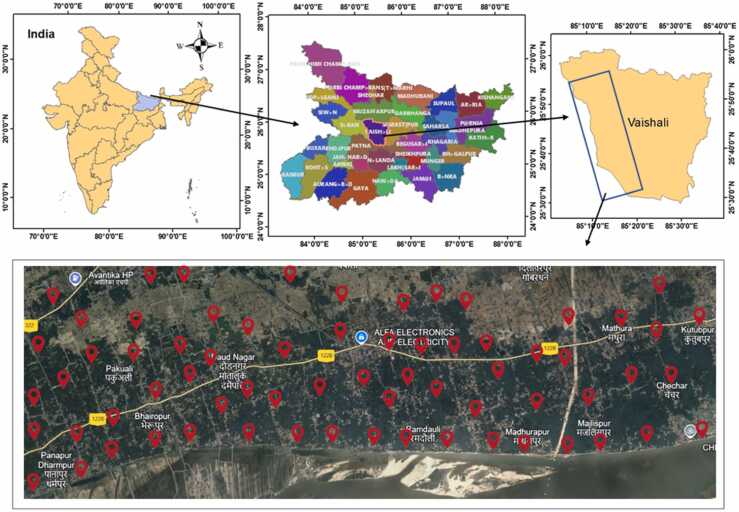
Map of study area (Bidupur Block, Vaishali district, Bihar, India) and groundwater sampling sites.

The Bidupur block is primarily an agricultural area having two main crops annually (wheat and rice) along with some vegetables. Moreover, this area is known as “the land of bananas” because of its large production and export throughout India. Groundwater is the primary source of water used in cultivation. The study area falls within the subtropical monsoon, having mild and dry winter and hot summer. It has deposits of arsenic-rich alluvium carried over by the river Ganga from the Himalayas [30]. Hence, there is a probability of the occurrence of arsenic in its groundwater due to the high percolation capacity of alluvial soil present in this area [59], [60]. Consequently, the cultivation of most of the agricultural crops depends upon groundwater which further contributes to arsenic exposures in humans [39]. Fig. 1 shows the location of the study area and sampling sites within the state of Bihar (India).

### 2.2 Sample analysis

Groundwater samples were taken from 16 randomly selected villages in the Bidupur block of Vaishali, Bihar. A total of 68 groundwater samples, obtained from public and private tubewells, were analysed on the spot for the estimation of arsenic in them through MQuant arsenic test kit (Merck, Germany) using colorimetric method with test strips and reagents (Range: 0.005 – 0.010 – 0.025 – 0.05 – 0.10 – 0.25 – 0.50 mg/l As). The groundwater sources under study were primarily used for drinking, cooking and irrigation purposes. The arsenic measurement was carried out as per the instructions provided in the test kit manual.

### 2.3 Contamination factor (Cf) assessment

**Fig. 2:**
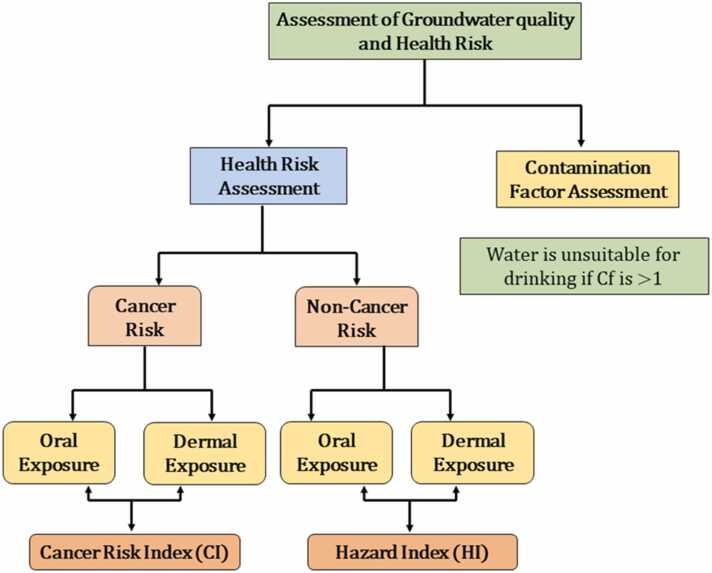
An overview of the assessment of groundwater quality and health risk.

The severity of groundwater contamination, which reflects water quality, was determined by calculating the contamination factor (Cf) followed by its comparison with contamination standard as specified by World Health Organization (WHO) and the Bureau of Indian Standards (BIS). In the present investigation, the level of groundwater arsenic contamination, expressed as contamination factor (Fig. 2), was evaluated using the following equation (1) [41], [61].

(1) $$\mathrm{Cf}=\;C_{i}/C_{o}$$

Where Cf means contamination factor, Ci represents the groundwater arsenic level, and Co reflects the contamination standard as specified by WHO and BIS [32]. The degree of groundwater contamination was categorized into safe (<1), moderate (1–3), high (3–6), and very high (>6) based on their contamination factor values, mentioned in parentheses [41], [62], [63].

### 2.4 Human health risk assessment

Health risk, defined as a threat caused by daily exposure of a toxic substance (such as arsenic) through oral and dermal routes, is assessed through the equations suggested by the US-EPA [29], [64], [65], [66]. Therefore, the estimation of health risk is crucial for the prediction of future health hazards caused by the exposure of toxic substances present in water. Health risk is further classified into two categories: non-cancer risk or hazard quotient (HQ) and cancer risk (CR).

#### 2.4.1 Exposure assessment

##### 2.4.1.1 Oral exposure

Assessment was done for individuals consuming arsenic through oral intake of groundwater. The average daily dose (ADD) of arsenic, if any, through drinking water was calculated using the USEPA equation (2) given below.

(2) $$\mathrm{ADD}=\frac{C\;\times \mathrm{IR}\times \mathrm{ED}\times \mathrm{EF}}{\mathrm{BW}\times \mathrm{AT}}$$

Where C = Concentration of arsenic in water (mg/L), IR = Intake rate (L/day), ED = Exposure duration equivalent to average lifespan of adult individuals, EF = Exposure frequency, BW = Body weight and AT= Average time (varies according to ED as AT = ED × EF). The values of above parameters have been mentioned in Table 1.

**Table 1:** Parameters for the assessment of oral and dermal exposures to groundwater arsenic in humans.

Parameters	Mode of Exposure	Value for Children	Value for Adults (males/ females)	Unit	Source
IR	Oral	0.64	2	l/day	[67]
ED	Oral/Dermal	12	68/70	years	[65]
EF	Oral	365	365	days/year	[68]
	Dermal	350	350	days/year	[69]
BW	Oral/Dermal	30	65/60	kg	CSIR-NIN, 2023
AT	Oral	12 × 365	65 × 365	days	[70]
	Dermal	18 × 200	60 × 200	days	[68]
SA	Dermal	6600	18,000	cm^2^	[65], [68]
EV	Dermal	1	1		[65]
K_p_	Dermal	10^−3^	10^−3^	cm/h	[65]
t_event_	Dermal	0.54	0.71	h/event	[67]

Abbreviations: IR = Intake rate, ED = Exposure duration, EF = Exposure frequency, AT = Average time, SA = Surface area, EV = Event frequency, Kp = Permeability coefficient, tevent = Duration of contact per event

##### 2.4.1.2 Dermal exposure

As arsenic can also enter the human body through skin when it comes in contact with groundwater during bathing, swimming and other household works [66]. Dermal absorbed dose (DAD) can be calculated by using the USEPA equation (3) given below.

(3) $$\mathrm{DA}D=\frac{\mathrm{DAevent}\times \mathrm{EV}\times \mathrm{ED}\times \mathrm{EF}\times \mathrm{SA}}{\mathrm{BW}\times \mathrm{AT}}$$

Where DAevent is the absorbed dose at every event of exposure (mg/cm^2^), SA represents the total surface area of skin available for contact (cm^2^), and EV is the event frequency of exposure per day. Values of ED (exposure duration), EF (exposure frequency), BW (body weight) and AT (average time) are mentioned in Table 1.

DAevent is calculated by the given equation (4) suggested by USEPA [66].

(4) DAevent=Kp×Cw×tevent

Where Kp is the dermal permeability coefficient of arsenic in groundwater (cm/h), Cw refers the concentration of arsenic in the groundwater (mg/l), and tevent is the duration of a single contact event (h).

#### 2.4.2 Risk assessment

##### 2.4.2.1 Non-cancer risk (hazard quotient)

Non-cancer risk was determined among the individuals of the study area through the determination of hazard quotient for both oral (HQoral) and dermal (HQdermal) exposures, using the equation (5).

$${\mathrm{HQ}}_{\mathrm{Oral}\;}=\;\mathrm{ADD}/\mathrm{RfD}$$

(5) $${\mathrm{HQ}}_{\mathrm{Dermal}}=\;\mathrm{ADD}/\mathrm{RfD}$$

Where RfD represents oral and dermal reference dose (0.0003 and 0.000123 mg/kg bw/day respectively) as per US-EPA (1998) guidelines [25].

The hazard index (HI) was then calculated by the addition of oral and dermal hazard quotients.

$$\mathrm{HI}={\mathrm{HQ}}_{\mathrm{Oral}}+{\mathrm{HQ}}_{\mathrm{Dermal}}$$

The threshold value for hazard index (HI) as per USEPA is 1. If the value of HI is above 1, it indicates the existence of non-cancerous health hazards [69].

##### 2.4.2.2 Cancer risk

Cancer risk (CR) was then calculated by using the given equation (6).

$${\mathrm{CR}}_{\mathrm{Oral}}=\mathrm{ADD}/\mathrm{CSF}$$

(6) $${\mathrm{CR}}_{\mathrm{Dermal}}=\;\mathrm{ADD}/\mathrm{CSF}$$

Where CSF is the cancer slope factor for oral and dermal exposures to arsenic and their values are 1.5 and 3.66 mg/kg bw/day [25], [71].

The total potential cancer risk or cancer index (CI) induced by arsenic was then calculated by adding oral and dermal cancer risks together [64] as per the given formula.

$$\mathrm{CI}=\;{\mathrm{CI}}_{\mathrm{Oral}}+\;{\mathrm{CI}}_{\mathrm{Dermal}\;}$$

The acceptable value of cancer index caused by arsenic exposure is 10^−6^ to 10^−4^ [66]. The cancer risk is considered high, if the value is greater than 10^−4^.

## 3 Results and discussion

### 3.1 Groundwater arsenic contamination

Exposure to arsenic through groundwater in humans poses a significant impact on human health. Thus, regular monitoring of arsenic levels in groundwater and risk assessment of its exposure on human health is highly pertinent. This study pertains to the quantification of the arsenic level in groundwater followed by its risk assessment on human health in the Vaishali district of Bihar (India).

**Fig. 3:**
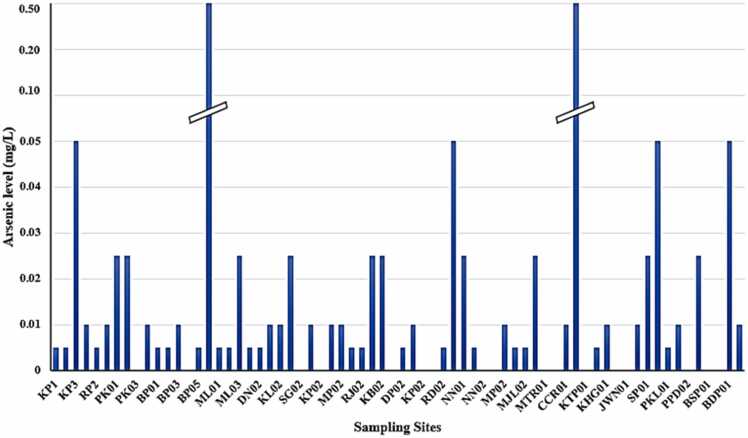
Groundwater arsenic levels at various sampling locations within the study area.

A total of 68 groundwater samples (in duplicate) were collected from the study area in which arsenic level ranged from 0 to its limit of 0.500 mg/l (Fig. 3). The average arsenic level and its standard deviation of mean were 0.025 mg/l and 0.084 mg/l respectively which showed the existence of large-scale variation in arsenic concentration in ground waters of different locations in the study area. About 25 % groundwater samples had nil (0 mg/l) arsenic level, while 27.94 % and 23.53 % samples had maximum arsenic levels of 0.005 mg/l, and 0.010 mg/l respectively, showing that 76.47 % of groundwater samples had arsenic concentrations within the WHO permissible limit . Among the remaining groundwater samples, 14.71 % and 5.88 % samples had 0.025 mg/l and 0.050 mg/l arsenic levels respectively. Moreover, 2.94 % of groundwater samples (Sample Id-HN01 and CCR02) exceeded the 0.500 mg/l limit of arsenic content. Cumulatively, 3/4th of the samples were within the WHO permissible limit, and only 1/4th of the samples exceeded the limit. The possible cause of arsenic contamination in the study area is the high percolation capacity of alluvial sediments which causes the reductive dissolution of hydrated iron oxides present in Holocene deposits, leading to the release of arsenic in groundwater [57], [72]. A strong association has also been identified between the over-extraction of groundwater for household and agricultural purposes and the levels of arsenic content [73]. However, the results shown in this study were less than previous studies carried out in the same region of Bihar [6], [7].

### 3.2 Contamination factor assessment

**Fig. 4:**
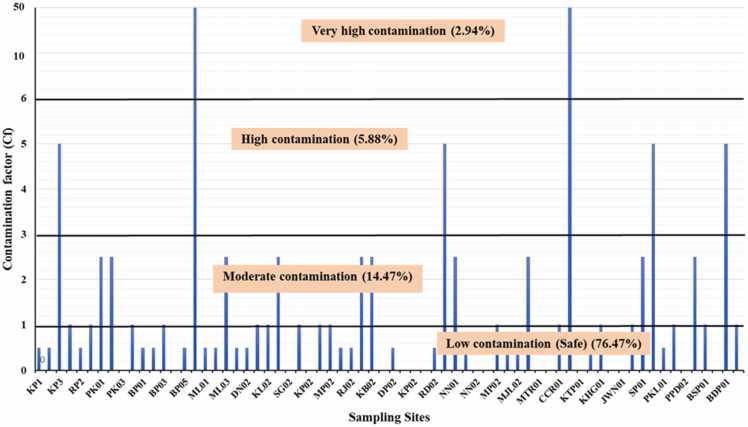
Contamination factors and index of groundwater arsenic contamination.

The assessment of the suitability of groundwater for drinking and other purposes was done by calculating the contamination factor for the evaluation of water quality using the Eq. (1) mentioned earlier. The results were ranging from 0 (minimum) to 50 (maximum) with an average value of 2.50. Water is said to be contaminated when its value is greater than 1. The results of this study have been shown in Fig. 4. The results showed that 76.47 % of groundwater samples were found safe for drinking and other purposes, while 23.53 % samples had crossed the limit which was considered unsuitable for drinking. Among about 24 % samples with less contamination level, about 15 % had moderate levels of contamination, about 6 % had high levels of contamination and only about 3 % samples (HN01 and CCR02) exhibited an exceptionally high level of contamination. These values were, however, slightly different from those reported previously at some places in India and abroad [6], [27], [31], [74].

### 3.3 Human health risk assessment

The average daily doses of arsenic through intake of groundwater for different age groups as well as sex through oral and dermal routes were calculated. The results showed that the average daily doses (ADDoral) of arsenic through the oral route for adult individuals (adult female 8.35 × 10^−4^ mg/kg/day; adult male 7.71 × 10^−4^ mg/kg/day) were significantly higher than those of children (5.30 × 10^−4^ mg/kg/day). The dermal average daily doses (ADDdermal) of arsenic for adult individuals (adult females 5.34 × 10^−5^ mg/kg/day; adult males 4.92 × 10^−5^ mg/kg/day) were also higher than those of children (1.57 × 10^−5^ mg/kg/day). The higher ADD for both oral and dermal routes in adults compared to children was due to higher intake rate of groundwater coupled with longer duration of exposure in adults.

### 3.4 Non-cancer risk assessment

**Fig. 5:**
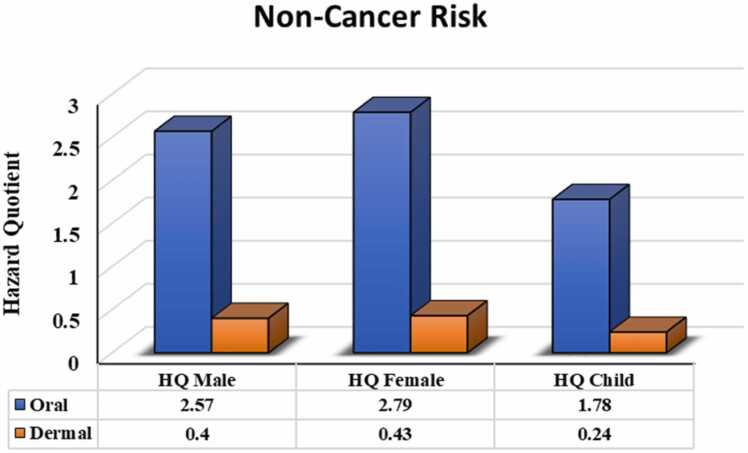
Oral and dermal non-cancer risks (hazard quotient) among different age groups.

Health risks other than cancer among the exposed population primarily depend upon the level of groundwater arsenic as well as the amount of water intake per day. Other affecting factors include body weight and exposure duration. If HQ is more than 1, the health risk (non-cancer risk) is very high. In our findings, the oral hazard quotient of adult females, adult males and children were 2.78, 2.57 and 1.78, respectively. Similarly, the dermal hazard quotient for adult females, adult males, and children were 0.43, 0.40 and 0.24, respectively. These values are greater than the previous study of Patel et al. [64]. The oral hazard quotient for all age groups exceeded the standard value (Fig. 5), but the dermal hazard quotient was below its threshold value. Hence, the oral intake of arsenic-containing groundwater may cause several health hazards, while dermal contact appears to be out of danger. The cumulative hazard quotient, called the hazard index (HI), was 3.21, 2.97, and 2.02 for adult females, adult males and children, respectively, which exceeded their acceptable limits. A similar trend with higher HI in adult females and adult males compared to children was found in the study of Moghaddam et al. [56]. Several researchers have also reported the occurrence of various clinical or subclinical complications in individuals upon long-term exposure to low levels of arsenic [22], [75], [76], [77], [78], [79], [80], [81], [82], [83], [84].

### 3.5 Cancer risk assessment

The cancer risk (CR) indicates the probability of having cancer in the population through the intake of toxic substances. Such a risk appears to be insignificant if its value is less than 1.0 × 10^–6^ (acceptable range 1.0 ×10^–6^ to 1.0 ×10^–4^) [70], [85].

**Fig. 6:**
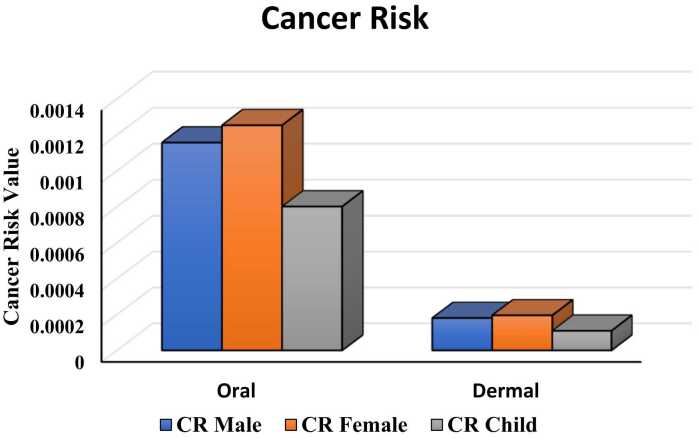
Cancer risk through oral and dermal exposure to groundwater arsenic among different age groups.

Based upon the oral intake of arsenic, the values for cancer risk were 1.25 × 10^−3^, 1.16 × 10^−3^ and 0.80 × 10^−3^ for adult females, adult males and children, respectively, which were 10–1000 times higher than the acceptable limit. In contrast, the values based on dermal exposure of arsenic for cancer risk were 1.95 × 10^−4^, 1.80 × 10^−4^ and 1.09 × 10^−4^ for adult females, adult males and children respectively, which were slightly higher than the acceptable limit, so the involvement of dermal exposure in carcinogenesis cannot be ruled out (Fig. 6, Table 2).

**Table 2:** Average ADD, HQ, HI, CR, and CI through both Oral and Dermal exposure to arsenic-contaminated groundwater.

Age Group	ADD		HQ		HI	CR		CI
	**Oral**	**Dermal**	**Oral**	**Dermal**		**Oral**	**Dermal**	
Adult Male	7.71 × 10^−4^	4.92 × 10^−5^	2.57	0.40	2.97	1.16 × 10^−3^	1.80 × 10^−4^	1.33 × 10^−3^
Adult Female	8.35 × 10^−4^	5.34 × 10^−5^	2.78	0.43	3.21	1.25 × 10^−3^	1.95 × 10^−4^	1.45 × 10^−3^
Children	5.30 × 10^−4^	2.98 × 10^−5^	1.78	0.24	2.02	0.80 × 10^−3^	1.09 × 10^−4^	0.91 × 10^−3^

The total potential cancer risk or cancer index (CI) through both oral and dermal exposures of arsenic was 1.45 × 10^−3^, 1.33 × 10^−3^ and 0.91 × 10^−3^ for adult females, adult males and children, respectively. As the values of both CR and CI surpass the acceptable limit of USEPA [70], suggesting that inhabitants of the study area are at higher risk of cancer. Previous observations have also shown the existence of cancer risk even at low levels of arsenic, capable of inducing human cancers [22], [27], [75], [86], [87]. Prolonged oral intake along with minimal dermal exposure causes a lifetime cancer risk in the inhabitants [88], [89].

The findings of the present work, therefore, exhibit deviation from the general trend and specifically report the less probability of non-cancer and cancer risks among the children compared to adults. This might be due to comparatively low intake rate and shorter period of exposure to arsenic in children than adults.

## 4 Conclusion

The present investigation, initially dealing with the quantification of groundwater arsenic in the Bidupur block of Vaishali district, Bihar (India), revealed that only one-fourth of collected samples had arsenic levels more than the WHO permissible limit. Assessment of the contamination factor further revealed the presence of poor water quality in almost equal number of samples. The average daily dose of arsenic through groundwater following oral and dermal routes was found higher among adult females compared to adult males and lower among children. Consequently, the cumulative hazard quotient or hazard index (non-cancer risk) was highest among adult females then adult males and lowest among children. Similarly, the probability of cancer risk was also highest among adult females than adult males and lowest among children. The underlying reason for the existence of lower health risk among children compared to adults might be due to their lesser rate of water intake and shorter period of exposure to arsenic.

Arsenic, therefore, poses high non-cancer and cancer risks even at its low levels of exposure. However, more extensive study is needed to reach at a broader conclusion. Again, monitoring of groundwater arsenic is highly advisable at regular interval to screen the health risks of individuals.

## CRediT authorship contribution statement

**Amod Kumar:** Writing – review & editing, Validation, Formal analysis. **Parimal Kumar Khan:** Writing – review & editing, Validation, Supervision, Conceptualization. **Pankaj Kumar:** Writing – original draft, Visualization, Software, Methodology, Investigation, Data curation, Conceptualization.

## Funding

The authors did not get any financial support for this study.

## Declaration of Competing Interest

The authors declare that they have no known competing financial interests or personal relationships that could have appeared to influence the work reported in this paper.

## Data Availability

Data will be made available on request.
