# Supplementary material for: Health risk assessment upon exposure to groundwater arsenic among individuals of different sex and age groups of Vaishali district, Bihar (India)
Source: Toxicol Rep. 2025 Apr 9;14:102024. doi: 10.1016/j.toxrep.2025.102024 (PMC12049810; doi:10.1016/j.toxrep.2025.102024)
Supplement: Supplementary file 1 — Supplementary material [file mmc1.docx]

Table 1: Arsenic contamination levels based on measured concentration (Cᵢ) and Cᵢᶠ values, indicating safety and risk severity.

| **Measured Arsenic (Cᵢ) (mg/L)** | **Cᵢᶠ Value** | **Contamination Level** |
| --- | --- | --- |
| ≤ 0.01 | ≤ 1 | Low contamination (Safe) |
| 0.01 - 0.03 | 1 - 3 | Moderate contamination |
| 0.03 - 0.06 | 3 - 6 | High contamination |
| > 0.06 | >6 | Very high contamination |

Table 2: Water quality index percentage with respect to different contamination level

| **Contamination Level** | **WQI Percentage** |
| --- | --- |
| Low contamination (Safe) | 76.47 |
| Moderate contamination | 14.70 |
| High contamination | 5.88 |
| Very high contamination | 2.94 |

Fig. 1. Percentage of WQI

Table 3. Water quality index (WQI) classification for the individual water samples

|  | | | |
| --- | --- | --- | --- |
| **Sl. No.** | **Sample Id** | **WQI** | **Contamination Level** |
| 1 | KP1 | 0.5 | Low contamination (Safe) |
| 2 | KP2 | 0.5 | Low contamination (Safe) |
| 3 | KP3 | 5 | Low contamination (Safe) |
| 4 | RP1 | 1 | Low contamination (Safe) |
| 5 | RP2 | 0.5 | Low contamination (Safe) |
| 6 | RP3 | 1 | Low contamination (Safe) |
| 7 | PK01 | 2.5 | Moderate contamination |
| 8 | PK02 | 2.5 | Moderate contamination |
| 9 | PK03 | 0 | Low contamination (Safe) |
| 10 | PK04 | 1 | Low contamination (Safe) |
| 11 | BP01 | 0.5 | Low contamination (Safe) |
| 12 | BP02 | 0.5 | Low contamination (Safe) |
| 13 | BP03 | 1 | Low contamination (Safe) |
| 14 | BP04 | 0 | Low contamination (Safe) |
| 15 | BP05 | 0.5 | Low contamination (Safe) |
| 16 | HN01 | 50 | Very high contamination |
| 17 | ML01 | 0.5 | Low contamination (Safe) |
| 18 | ML02 | 0.5 | Low contamination (Safe) |
| 19 | ML03 | 2.5 | Low contamination (Safe) |
| 20 | DN01 | 0.5 | Low contamination (Safe) |
| 21 | DN02 | 0.5 | Low contamination (Safe) |
| 22 | KL01 | 1 | Low contamination (Safe) |
| 23 | KL02 | 1 | Low contamination (Safe) |
| 24 | SG01 | 2.5 | Moderate contamination |
| 25 | SG02 | 0 | Low contamination (Safe) |
| 26 | KP01 | 1 | Low contamination (Safe) |
| 27 | KP02 | 0 | Low contamination (Safe) |
| 28 | MP01 | 1 | Low contamination (Safe) |
| 29 | MP02 | 1 | Low contamination (Safe) |
| 30 | RJ01 | 0.5 | Low contamination (Safe) |
| 31 | RJ02 | 0.5 | Low contamination (Safe) |
| 32 | KB01 | 2.5 | Moderate contamination |
| 33 | KB02 | 2.5 | Moderate contamination |
| 34 | DP01 | 0 | Low contamination (Safe) |
| 35 | DP02 | 0.5 | Low contamination (Safe) |
| 36 | KP01 | 0 | Low contamination (Safe) |
| 37 | KP02 | 0 | Low contamination (Safe) |
| 38 | RD01 | 0 | Low contamination (Safe) |
| 39 | RD02 | 0.5 | Low contamination (Safe) |
| 40 | AM01 | 5 | High contamination |
| 41 | NN01 | 2.5 | Moderate contamination |
| 42 | MP01 | 0.5 | Low contamination (Safe) |
| 43 | NN02 | 0 | Low contamination (Safe) |
| 44 | BP01 | 0 | Low contamination (Safe) |
| 45 | MP02 | 1 | Low contamination (Safe) |
| 46 | MJL01 | 0.5 | Low contamination (Safe) |
| 47 | MJL02 | 0.5 | Low contamination (Safe) |
| 48 | GPL01 | 2.5 | Moderate contamination |
| 49 | MTR01 | 0 | Low contamination (Safe) |
| 50 | GPL02 | 0 | Low contamination (Safe) |
| 51 | CCR01 | 1 | Low contamination (Safe) |
| 52 | CCR02 | 50 | Very high contamination |
| 53 | KTP01 | 0 | Low contamination (Safe) |
| 54 | BJP01 | 0.5 | Low contamination (Safe) |
| 55 | KHG01 | 1 | Low contamination (Safe) |
| 56 | GBP01 | 0 | Low contamination (Safe) |
| 57 | JWN01 | 0 | Low contamination (Safe) |
| 58 | HNM01 | 1 | Low contamination (Safe) |
| 59 | SP01 | 2.5 | Moderate contamination |
| 60 | CKS01 | 5 | High contamination |
| 61 | PKL01 | 0.5 | Low contamination (Safe) |
| 62 | PPD01 | 1 | Low contamination (Safe) |
| 63 | PPD02 | 0 | Low contamination (Safe) |
| 64 | DLP01 | 2.5 | Moderate contamination |
| 65 | BSP01 | 1 | Low contamination (Safe) |
| 66 | KTP02 | 0 | Low contamination (Safe) |
| 67 | BDP01 | 5 | High contamination |
| 68 | SDP01 | 1 | Low contamination (Safe) |
|  | **Mean** | **2.507353** |  |

Table 3: Non-Cancer Risk or Hazardous Quotient (HQ) through oral and dermal intake of groundwater among male, female and child

|  | HQ Male | HQ Female | HQ Child |
| --- | --- | --- | --- |
| Oral | 2.57 | 2.79 | 1.78 |
| Dermal | 0.4 | 0.43 | 0.24 |
|  |  |  |  |
| SE oral | 1.047 | 1.135 | 0.726 |
| SE dermal | 0.1633 | 0.1769 | 0.0986 |

Table 4: Cancer Risk through oral and dermal intake of groundwater among male, female and child

|  | **CR Male** | **CR Female** | **CR Child** |
| --- | --- | --- | --- |
| Oral | 0.00115724 | 0.001253676 | 0.000802353 |
| Dermal | 0.000180432 | 0.000195468 | 0.000109022 |
| SEO | 0.000419153 | 0.000454083 | 0.000227041 |
| SED | 5.4885E-06 | 5.94588E-06 | 3.31629E-06 |

| **Calculate Average Daily Dose (ADD), Hazard Quotient (HQ), and Cancer Risk (CR) using Arsenic conc. in the groundwater (For Adult Male)** | | | | | | | | | | | |
| --- | --- | --- | --- | --- | --- | --- | --- | --- | --- | --- | --- |
| **Sampling Site** | **Arsenic level (mg/l)** | **Exposure duration (Years)** | **Intake rate (L/day)** | **Exposure Frequency (Days/Year)** | **Body Weight (Kg)** | **Average time (Days)** | **Average daily dose (mg/kg/day)** | **Reference Dose (mg/kg/day)** | **Hazard Quotient (HQ)** | **Cancer Slope Factor (mg/kg/day)** | **Cancer Risk (CR)** |
| KP1 | 0.005 | 68 | 2 | 365 | 65 | 24820 | 0.00015 | 0.0003 | 0.512820513 | 1.5 | 0.000230769 |
| KP2 | 0.005 | 68 | 2 | 365 | 65 | 24820 | 0.00015 | 0.0003 | 0.512820513 | 1.5 | 0.000230769 |
| KP3 | 0.05 | 68 | 2 | 365 | 65 | 24820 | 0.00154 | 0.0003 | 5.128205128 | 1.5 | 0.002307692 |
| RP1 | 0.01 | 68 | 2 | 365 | 65 | 24820 | 0.00031 | 0.0003 | 1.025641026 | 1.5 | 0.000461538 |
| RP2 | 0.005 | 68 | 2 | 365 | 65 | 24820 | 0.00015 | 0.0003 | 0.512820513 | 1.5 | 0.000230769 |
| RP3 | 0.01 | 68 | 2 | 365 | 65 | 24820 | 0.00031 | 0.0003 | 1.025641026 | 1.5 | 0.000461538 |
| PK01 | 0.025 | 68 | 2 | 365 | 65 | 24820 | 0.00077 | 0.0003 | 2.564102564 | 1.5 | 0.001153846 |
| PK02 | 0.025 | 68 | 2 | 365 | 65 | 24820 | 0.00077 | 0.0003 | 2.564102564 | 1.5 | 0.001153846 |
| PK03 | 0 | 68 | 2 | 365 | 65 | 24820 | 0.00000 | 0.0003 | 0 | 1.5 | 0 |
| PK04 | 0.01 | 68 | 2 | 365 | 65 | 24820 | 0.00031 | 0.0003 | 1.025641026 | 1.5 | 0.000461538 |
| BP01 | 0.005 | 68 | 2 | 365 | 65 | 24820 | 0.00015 | 0.0003 | 0.512820513 | 1.5 | 0.000230769 |
| BP02 | 0.005 | 68 | 2 | 365 | 65 | 24820 | 0.00015 | 0.0003 | 0.512820513 | 1.5 | 0.000230769 |
| BP03 | 0.01 | 68 | 2 | 365 | 65 | 24820 | 0.00031 | 0.0003 | 1.025641026 | 1.5 | 0.000461538 |
| BP04 | 0 | 68 | 2 | 365 | 65 | 24820 | 0.00000 | 0.0003 | 0 | 1.5 | 0 |
| BP05 | 0.005 | 68 | 2 | 365 | 65 | 24820 | 0.00015 | 0.0003 | 0.512820513 | 1.5 | 0.000230769 |
| HN01 | 0.5 | 68 | 2 | 365 | 65 | 24820 | 0.01538 | 0.0003 | 51.28205128 | 1.5 | 0.023076923 |
| ML01 | 0.005 | 68 | 2 | 365 | 65 | 24820 | 0.00015 | 0.0003 | 0.512820513 | 1.5 | 0.000230769 |
| ML02 | 0.005 | 68 | 2 | 365 | 65 | 24820 | 0.00015 | 0.0003 | 0.512820513 | 1.5 | 0.000230769 |
| ML03 | 0.025 | 68 | 2 | 365 | 65 | 24820 | 0.00077 | 0.0003 | 2.564102564 | 1.5 | 0.001153846 |
| DN01 | 0.005 | 68 | 2 | 365 | 65 | 24820 | 0.00015 | 0.0003 | 0.512820513 | 1.5 | 0.000230769 |
| DN02 | 0.005 | 68 | 2 | 365 | 65 | 24820 | 0.00015 | 0.0003 | 0.512820513 | 1.5 | 0.000230769 |
| KL01 | 0.01 | 68 | 2 | 365 | 65 | 24820 | 0.00031 | 0.0003 | 1.025641026 | 1.5 | 0.000461538 |
| KL02 | 0.01 | 68 | 2 | 365 | 65 | 24820 | 0.00031 | 0.0003 | 1.025641026 | 1.5 | 0.000461538 |
| SG01 | 0.025 | 68 | 2 | 365 | 65 | 24820 | 0.00077 | 0.0003 | 2.564102564 | 1.5 | 0.001153846 |
| SG02 | 0 | 68 | 2 | 365 | 65 | 24820 | 0.00000 | 0.0003 | 0 | 1.5 | 0 |
| KP01 | 0.01 | 68 | 2 | 365 | 65 | 24820 | 0.00031 | 0.0003 | 1.025641026 | 1.5 | 0.000461538 |
| KP02 | 0 | 68 | 2 | 365 | 65 | 24820 | 0.00000 | 0.0003 | 0 | 1.5 | 0 |
| MP01 | 0.01 | 68 | 2 | 365 | 65 | 24820 | 0.00031 | 0.0003 | 1.025641026 | 1.5 | 0.000461538 |
| MP02 | 0.01 | 68 | 2 | 365 | 65 | 24820 | 0.00031 | 0.0003 | 1.025641026 | 1.5 | 0.000461538 |
| RJ01 | 0.005 | 68 | 2 | 365 | 65 | 24820 | 0.00015 | 0.0003 | 0.512820513 | 1.5 | 0.000230769 |
| RJ02 | 0.005 | 68 | 2 | 365 | 65 | 24820 | 0.00015 | 0.0003 | 0.512820513 | 1.5 | 0.000230769 |
| KB01 | 0.025 | 68 | 2 | 365 | 65 | 24820 | 0.00077 | 0.0003 | 2.564102564 | 1.5 | 0.001153846 |
| KB02 | 0.025 | 68 | 2 | 365 | 65 | 24820 | 0.00077 | 0.0003 | 2.564102564 | 1.5 | 0.001153846 |
| DP01 | 0 | 68 | 2 | 365 | 65 | 24820 | 0.00000 | 0.0003 | 0 | 1.5 | 0 |
| DP02 | 0.005 | 68 | 2 | 365 | 65 | 24820 | 0.00015 | 0.0003 | 0.512820513 | 1.5 | 0.000230769 |
| KP01 | 0 | 68 | 2 | 365 | 65 | 24820 | 0.00000 | 0.0003 | 0 | 1.5 | 0 |
| KP02 | 0 | 68 | 2 | 365 | 65 | 24820 | 0.00000 | 0.0003 | 0 | 1.5 | 0 |
| RD01 | 0 | 68 | 2 | 365 | 65 | 24820 | 0.00000 | 0.0003 | 0 | 1.5 | 0 |
| RD02 | 0.005 | 68 | 2 | 365 | 65 | 24820 | 0.00015 | 0.0003 | 0.512820513 | 1.5 | 0.000230769 |
| AM01 | 0.05 | 68 | 2 | 365 | 65 | 24820 | 0.00154 | 0.0003 | 5.128205128 | 1.5 | 0.002307692 |
| NN01 | 0.025 | 68 | 2 | 365 | 65 | 24820 | 0.00077 | 0.0003 | 2.564102564 | 1.5 | 0.001153846 |
| MP01 | 0.005 | 68 | 2 | 365 | 65 | 24820 | 0.00015 | 0.0003 | 0.512820513 | 1.5 | 0.000230769 |
| NN02 | 0 | 68 | 2 | 365 | 65 | 24820 | 0.00000 | 0.0003 | 0 | 1.5 | 0 |
| BP01 | 0 | 68 | 2 | 365 | 65 | 24820 | 0.00000 | 0.0003 | 0 | 1.5 | 0 |
| MP02 | 0.01 | 68 | 2 | 365 | 65 | 24820 | 0.00031 | 0.0003 | 1.025641026 | 1.5 | 0.000461538 |
| MJL01 | 0.005 | 68 | 2 | 365 | 65 | 24820 | 0.00015 | 0.0003 | 0.512820513 | 1.5 | 0.000230769 |
| MJL02 | 0.005 | 68 | 2 | 365 | 65 | 24820 | 0.00015 | 0.0003 | 0.512820513 | 1.5 | 0.000230769 |
| GPL01 | 0.025 | 68 | 2 | 365 | 65 | 24820 | 0.00077 | 0.0003 | 2.564102564 | 1.5 | 0.001153846 |
| MTR01 | 0 | 68 | 2 | 365 | 65 | 24820 | 0.00000 | 0.0003 | 0 | 1.5 | 0 |
| GPL02 | 0 | 68 | 2 | 365 | 65 | 24820 | 0.00000 | 0.0003 | 0 | 1.5 | 0 |
| CCR01 | 0.01 | 68 | 2 | 365 | 65 | 24820 | 0.00031 | 0.0003 | 1.025641026 | 1.5 | 0.000461538 |
| CCR02 | 0.5 | 68 | 2 | 365 | 65 | 24820 | 0.01538 | 0.0003 | 51.28205128 | 1.5 | 0.023076923 |
| KTP01 | 0 | 68 | 2 | 365 | 65 | 24820 | 0.00000 | 0.0003 | 0 | 1.5 | 0 |
| BJP01 | 0.005 | 68 | 2 | 365 | 65 | 24820 | 0.00015 | 0.0003 | 0.512820513 | 1.5 | 0.000230769 |
| KHG01 | 0.01 | 68 | 2 | 365 | 65 | 24820 | 0.00031 | 0.0003 | 1.025641026 | 1.5 | 0.000461538 |
| GBP01 | 0 | 68 | 2 | 365 | 65 | 24820 | 0.00000 | 0.0003 | 0 | 1.5 | 0 |
| JWN01 | 0 | 68 | 2 | 365 | 65 | 24820 | 0.00000 | 0.0003 | 0 | 1.5 | 0 |
| HNM01 | 0.01 | 68 | 2 | 365 | 65 | 24820 | 0.00031 | 0.0003 | 1.025641026 | 1.5 | 0.000461538 |
| SP01 | 0.025 | 68 | 2 | 365 | 65 | 24820 | 0.00077 | 0.0003 | 2.564102564 | 1.5 | 0.001153846 |
| CKS01 | 0.05 | 68 | 2 | 365 | 65 | 24820 | 0.00154 | 0.0003 | 5.128205128 | 1.5 | 0.002307692 |
| PKL01 | 0.005 | 68 | 2 | 365 | 65 | 24820 | 0.00015 | 0.0003 | 0.512820513 | 1.5 | 0.000230769 |
| PPD01 | 0.01 | 68 | 2 | 365 | 65 | 24820 | 0.00031 | 0.0003 | 1.025641026 | 1.5 | 0.000461538 |
| PPD02 | 0 | 68 | 2 | 365 | 65 | 24820 | 0.00000 | 0.0003 | 0 | 1.5 | 0 |
| DLP01 | 0.025 | 68 | 2 | 365 | 65 | 24820 | 0.00077 | 0.0003 | 2.564102564 | 1.5 | 0.001153846 |
| BSP01 | 0.01 | 68 | 2 | 365 | 65 | 24820 | 0.00031 | 0.0003 | 1.025641026 | 1.5 | 0.000461538 |
| KTP02 | 0 | 68 | 2 | 365 | 65 | 24820 | 0.00000 | 0.0003 | 0 | 1.5 | 0 |
| BDP01 | 0.05 | 68 | 2 | 365 | 65 | 24820 | 0.00154 | 0.0003 | 5.128205128 | 1.5 | 0.002307692 |
| SDP01 | 0.01 | 68 | 2 | 365 | 65 | 24820 | 0.00031 | 0.0003 | 1.025641026 | 1.5 | 0.000461538 |
|  |  |  |  |  |  |  |  |  |  |  |  |
|  |  |  |  |  |  | **Average** | **0.00077** |  | **2.571644042** |  | **0.00115724** |

| **Calculation of Average Daily Dose (ADD), Hazard Quotient (HQ), and Cancer Risk (CR) using Arsenic conc. in the groundwater (For Children)** | | | | | | | | | | | |
| --- | --- | --- | --- | --- | --- | --- | --- | --- | --- | --- | --- |
| **Sampling Site** | **Arsenic level (mg/l)** | **Exposure duration (Years)** | **Intake rate (L/day)** | **Exposure Frequency (Days/Year)** | **Body Weight (Kg)** | **Average time (Days)** | **Average daily dose (mg/kg/day)** | **Reference Dose (mg/kg/day)** | **Hazard Quotient (HQ)** | **Cancer Slope Factor (mg/kg/day)** | **Cancer Risk (CR)** |
| KP1 | 0.005 | 12 | 0.64 | 365 | 30 | 4380 | 0.00011 | 0.0003 | 0.355555556 | 1.5 | 0.00016 |
| KP2 | 0.005 | 12 | 0.64 | 365 | 30 | 4380 | 0.00011 | 0.0003 | 0.355555556 | 1.5 | 0.00016 |
| KP3 | 0.05 | 12 | 0.64 | 365 | 30 | 4380 | 0.00107 | 0.0003 | 3.555555556 | 1.5 | 0.0016 |
| RP1 | 0.01 | 12 | 0.64 | 365 | 30 | 4380 | 0.00021 | 0.0003 | 0.711111111 | 1.5 | 0.00032 |
| RP2 | 0.005 | 12 | 0.64 | 365 | 30 | 4380 | 0.00011 | 0.0003 | 0.355555556 | 1.5 | 0.00016 |
| RP3 | 0.01 | 12 | 0.64 | 365 | 30 | 4380 | 0.00021 | 0.0003 | 0.711111111 | 1.5 | 0.00032 |
| PK01 | 0.025 | 12 | 0.64 | 365 | 30 | 4380 | 0.00053 | 0.0003 | 1.777777778 | 1.5 | 0.0008 |
| PK02 | 0.025 | 12 | 0.64 | 365 | 30 | 4380 | 0.00053 | 0.0003 | 1.777777778 | 1.5 | 0.0008 |
| PK03 | 0 | 12 | 0.64 | 365 | 30 | 4380 | 0.00000 | 0.0003 | 0 | 1.5 | 0 |
| PK04 | 0.01 | 12 | 0.64 | 365 | 30 | 4380 | 0.00021 | 0.0003 | 0.711111111 | 1.5 | 0.00032 |
| BP01 | 0.005 | 12 | 0.64 | 365 | 30 | 4380 | 0.00011 | 0.0003 | 0.355555556 | 1.5 | 0.00016 |
| BP02 | 0.005 | 12 | 0.64 | 365 | 30 | 4380 | 0.00011 | 0.0003 | 0.355555556 | 1.5 | 0.00016 |
| BP03 | 0.01 | 12 | 0.64 | 365 | 30 | 4380 | 0.00021 | 0.0003 | 0.711111111 | 1.5 | 0.00032 |
| BP04 | 0 | 12 | 0.64 | 365 | 30 | 4380 | 0.00000 | 0.0003 | 0 | 1.5 | 0 |
| BP05 | 0.005 | 12 | 0.64 | 365 | 30 | 4380 | 0.00011 | 0.0003 | 0.355555556 | 1.5 | 0.00016 |
| HN01 | 0.5 | 12 | 0.64 | 365 | 30 | 4380 | 0.01067 | 0.0003 | 35.55555556 | 1.5 | 0.016 |
| ML01 | 0.005 | 12 | 0.64 | 365 | 30 | 4380 | 0.00011 | 0.0003 | 0.355555556 | 1.5 | 0.00016 |
| ML02 | 0.005 | 12 | 0.64 | 365 | 30 | 4380 | 0.00011 | 0.0003 | 0.355555556 | 1.5 | 0.00016 |
| ML03 | 0.025 | 12 | 0.64 | 365 | 30 | 4380 | 0.00053 | 0.0003 | 1.777777778 | 1.5 | 0.0008 |
| DN01 | 0.005 | 12 | 0.64 | 365 | 30 | 4380 | 0.00011 | 0.0003 | 0.355555556 | 1.5 | 0.00016 |
| DN02 | 0.005 | 12 | 0.64 | 365 | 30 | 4380 | 0.00011 | 0.0003 | 0.355555556 | 1.5 | 0.00016 |
| KL01 | 0.01 | 12 | 0.64 | 365 | 30 | 4380 | 0.00021 | 0.0003 | 0.711111111 | 1.5 | 0.00032 |
| KL02 | 0.01 | 12 | 0.64 | 365 | 30 | 4380 | 0.00021 | 0.0003 | 0.711111111 | 1.5 | 0.00032 |
| SG01 | 0.025 | 12 | 0.64 | 365 | 30 | 4380 | 0.00053 | 0.0003 | 1.777777778 | 1.5 | 0.0008 |
| SG02 | 0 | 12 | 0.64 | 365 | 30 | 4380 | 0.00000 | 0.0003 | 0 | 1.5 | 0 |
| KP01 | 0.01 | 12 | 0.64 | 365 | 30 | 4380 | 0.00021 | 0.0003 | 0.711111111 | 1.5 | 0.00032 |
| KP02 | 0 | 12 | 0.64 | 365 | 30 | 4380 | 0.00000 | 0.0003 | 0 | 1.5 | 0 |
| MP01 | 0.01 | 12 | 0.64 | 365 | 30 | 4380 | 0.00021 | 0.0003 | 0.711111111 | 1.5 | 0.00032 |
| MP02 | 0.01 | 12 | 0.64 | 365 | 30 | 4380 | 0.00021 | 0.0003 | 0.711111111 | 1.5 | 0.00032 |
| RJ01 | 0.005 | 12 | 0.64 | 365 | 30 | 4380 | 0.00011 | 0.0003 | 0.355555556 | 1.5 | 0.00016 |
| RJ02 | 0.005 | 12 | 0.64 | 365 | 30 | 4380 | 0.00011 | 0.0003 | 0.355555556 | 1.5 | 0.00016 |
| KB01 | 0.025 | 12 | 0.64 | 365 | 30 | 4380 | 0.00053 | 0.0003 | 1.777777778 | 1.5 | 0.0008 |
| KB02 | 0.025 | 12 | 0.64 | 365 | 30 | 4380 | 0.00053 | 0.0003 | 1.777777778 | 1.5 | 0.0008 |
| DP01 | 0 | 12 | 0.64 | 365 | 30 | 4380 | 0.00000 | 0.0003 | 0 | 1.5 | 0 |
| DP02 | 0.005 | 12 | 0.64 | 365 | 30 | 4380 | 0.00011 | 0.0003 | 0.355555556 | 1.5 | 0.00016 |
| KP01 | 0 | 12 | 0.64 | 365 | 30 | 4380 | 0.00000 | 0.0003 | 0 | 1.5 | 0 |
| KP02 | 0 | 12 | 0.64 | 365 | 30 | 4380 | 0.00000 | 0.0003 | 0 | 1.5 | 0 |
| RD01 | 0 | 12 | 0.64 | 365 | 30 | 4380 | 0.00000 | 0.0003 | 0 | 1.5 | 0 |
| RD02 | 0.005 | 12 | 0.64 | 365 | 30 | 4380 | 0.00011 | 0.0003 | 0.355555556 | 1.5 | 0.00016 |
| AM01 | 0.05 | 12 | 0.64 | 365 | 30 | 4380 | 0.00107 | 0.0003 | 3.555555556 | 1.5 | 0.0016 |
| NN01 | 0.025 | 12 | 0.64 | 365 | 30 | 4380 | 0.00053 | 0.0003 | 1.777777778 | 1.5 | 0.0008 |
| MP01 | 0.005 | 12 | 0.64 | 365 | 30 | 4380 | 0.00011 | 0.0003 | 0.355555556 | 1.5 | 0.00016 |
| NN02 | 0 | 12 | 0.64 | 365 | 30 | 4380 | 0.00000 | 0.0003 | 0 | 1.5 | 0 |
| BP01 | 0 | 12 | 0.64 | 365 | 30 | 4380 | 0.00000 | 0.0003 | 0 | 1.5 | 0 |
| MP02 | 0.01 | 12 | 0.64 | 365 | 30 | 4380 | 0.00021 | 0.0003 | 0.711111111 | 1.5 | 0.00032 |
| MJL01 | 0.005 | 12 | 0.64 | 365 | 30 | 4380 | 0.00011 | 0.0003 | 0.355555556 | 1.5 | 0.00016 |
| MJL02 | 0.005 | 12 | 0.64 | 365 | 30 | 4380 | 0.00011 | 0.0003 | 0.355555556 | 1.5 | 0.00016 |
| GPL01 | 0.025 | 12 | 0.64 | 365 | 30 | 4380 | 0.00053 | 0.0003 | 1.777777778 | 1.5 | 0.0008 |
| MTR01 | 0 | 12 | 0.64 | 365 | 30 | 4380 | 0.00000 | 0.0003 | 0 | 1.5 | 0 |
| GPL02 | 0 | 12 | 0.64 | 365 | 30 | 4380 | 0.00000 | 0.0003 | 0 | 1.5 | 0 |
| CCR01 | 0.01 | 12 | 0.64 | 365 | 30 | 4380 | 0.00021 | 0.0003 | 0.711111111 | 1.5 | 0.00032 |
| CCR02 | 0.5 | 12 | 0.64 | 365 | 30 | 4380 | 0.01067 | 0.0003 | 35.55555556 | 1.5 | 0.016 |
| KTP01 | 0 | 12 | 0.64 | 365 | 30 | 4380 | 0.00000 | 0.0003 | 0 | 1.5 | 0 |
| BJP01 | 0.005 | 12 | 0.64 | 365 | 30 | 4380 | 0.00011 | 0.0003 | 0.355555556 | 1.5 | 0.00016 |
| KHG01 | 0.01 | 12 | 0.64 | 365 | 30 | 4380 | 0.00021 | 0.0003 | 0.711111111 | 1.5 | 0.00032 |
| GBP01 | 0 | 12 | 0.64 | 365 | 30 | 4380 | 0.00000 | 0.0003 | 0 | 1.5 | 0 |
| JWN01 | 0 | 12 | 0.64 | 365 | 30 | 4380 | 0.00000 | 0.0003 | 0 | 1.5 | 0 |
| HNM01 | 0.01 | 12 | 0.64 | 365 | 30 | 4380 | 0.00021 | 0.0003 | 0.711111111 | 1.5 | 0.00032 |
| SP01 | 0.025 | 12 | 0.64 | 365 | 30 | 4380 | 0.00053 | 0.0003 | 1.777777778 | 1.5 | 0.0008 |
| CKS01 | 0.05 | 12 | 0.64 | 365 | 30 | 4380 | 0.00107 | 0.0003 | 3.555555556 | 1.5 | 0.0016 |
| PKL01 | 0.005 | 12 | 0.64 | 365 | 30 | 4380 | 0.00011 | 0.0003 | 0.355555556 | 1.5 | 0.00016 |
| PPD01 | 0.01 | 12 | 0.64 | 365 | 30 | 4380 | 0.00021 | 0.0003 | 0.711111111 | 1.5 | 0.00032 |
| PPD02 | 0 | 12 | 0.64 | 365 | 30 | 4380 | 0.00000 | 0.0003 | 0 | 1.5 | 0 |
| DLP01 | 0.025 | 12 | 0.64 | 365 | 30 | 4380 | 0.00053 | 0.0003 | 1.777777778 | 1.5 | 0.0008 |
| BSP01 | 0.01 | 12 | 0.64 | 365 | 30 | 4380 | 0.00021 | 0.0003 | 0.711111111 | 1.5 | 0.00032 |
| KTP02 | 0 | 12 | 0.64 | 365 | 30 | 4380 | 0.00000 | 0.0003 | 0 | 1.5 | 0 |
| BDP01 | 0.05 | 12 | 0.64 | 365 | 30 | 4380 | 0.00107 | 0.0003 | 3.555555556 | 1.5 | 0.0016 |
| SDP01 | 0.01 | 12 | 0.64 | 365 | 30 | 4380 | 0.00021 | 0.0003 | 0.711111111 | 1.5 | 0.00032 |
|  |  |  |  |  |  |  |  |  |  |  |  |
|  |  |  |  |  |  | **Average** | **0.00053** |  | **1.783006536** |  | **0.000802353** |

| **Calculation of Average Daily Dose (ADD), Hazard Quotient (HQ), and Cancer Risk (CR) using Arsenic Conc. in the Groundwater (For Female)** | | | | | | | | | | | |
| --- | --- | --- | --- | --- | --- | --- | --- | --- | --- | --- | --- |
| **Sampling Site** | **Arsenic level (mg/l)** | **Exposure duration (Years)** | **Intake rate (L/day)** | **Exposure Frequency (Days/Year)** | **Body Weight (Kg)** | **Average time (Days)** | **Average daily dose (mg/kg/day)** | **Reference Dose (mg/kg/day)** | **Hazard Quotient (HQ)** | **Cancer Slope Factor (mg/kg/day)** | **Cancer Risk (CR)** |
| KP1 | 0.005 | 70 | 2 | 365 | 60 | 25550 | 0.00017 | 0.0003 | 0.555555556 | 1.5 | 0.00025 |
| KP2 | 0.005 | 70 | 2 | 365 | 60 | 25550 | 0.00017 | 0.0003 | 0.555555556 | 1.5 | 0.00025 |
| KP3 | 0.05 | 70 | 2 | 365 | 60 | 25550 | 0.00167 | 0.0003 | 5.555555556 | 1.5 | 0.0025 |
| RP1 | 0.01 | 70 | 2 | 365 | 60 | 25550 | 0.00033 | 0.0003 | 1.111111111 | 1.5 | 0.0005 |
| RP2 | 0.005 | 70 | 2 | 365 | 60 | 25550 | 0.00017 | 0.0003 | 0.555555556 | 1.5 | 0.00025 |
| RP3 | 0.01 | 70 | 2 | 365 | 60 | 25550 | 0.00033 | 0.0003 | 1.111111111 | 1.5 | 0.0005 |
| PK01 | 0.025 | 70 | 2 | 365 | 60 | 25550 | 0.00083 | 0.0003 | 2.777777778 | 1.5 | 0.00125 |
| PK02 | 0.025 | 70 | 2 | 365 | 60 | 25550 | 0.00083 | 0.0003 | 2.777777778 | 1.5 | 0.00125 |
| PK03 | 0 | 70 | 2 | 365 | 60 | 25550 | 0.00000 | 0.0003 | 0 | 1.5 | 0 |
| PK04 | 0.01 | 70 | 2 | 365 | 60 | 25550 | 0.00033 | 0.0003 | 1.111111111 | 1.5 | 0.0005 |
| BP01 | 0.005 | 70 | 2 | 365 | 60 | 25550 | 0.00017 | 0.0003 | 0.555555556 | 1.5 | 0.00025 |
| BP02 | 0.005 | 70 | 2 | 365 | 60 | 25550 | 0.00017 | 0.0003 | 0.555555556 | 1.5 | 0.00025 |
| BP03 | 0.01 | 70 | 2 | 365 | 60 | 25550 | 0.00033 | 0.0003 | 1.111111111 | 1.5 | 0.0005 |
| BP04 | 0 | 70 | 2 | 365 | 60 | 25550 | 0.00000 | 0.0003 | 0 | 1.5 | 0 |
| BP05 | 0.005 | 70 | 2 | 365 | 60 | 25550 | 0.00017 | 0.0003 | 0.555555556 | 1.5 | 0.00025 |
| HN01 | 0.5 | 70 | 2 | 365 | 60 | 25550 | 0.01667 | 0.0003 | 55.55555556 | 1.5 | 0.025 |
| ML01 | 0.005 | 70 | 2 | 365 | 60 | 25550 | 0.00017 | 0.0003 | 0.555555556 | 1.5 | 0.00025 |
| ML02 | 0.005 | 70 | 2 | 365 | 60 | 25550 | 0.00017 | 0.0003 | 0.555555556 | 1.5 | 0.00025 |
| ML03 | 0.025 | 70 | 2 | 365 | 60 | 25550 | 0.00083 | 0.0003 | 2.777777778 | 1.5 | 0.00125 |
| DN01 | 0.005 | 70 | 2 | 365 | 60 | 25550 | 0.00017 | 0.0003 | 0.555555556 | 1.5 | 0.00025 |
| DN02 | 0.005 | 70 | 2 | 365 | 60 | 25550 | 0.00017 | 0.0003 | 0.555555556 | 1.5 | 0.00025 |
| KL01 | 0.01 | 70 | 2 | 365 | 60 | 25550 | 0.00033 | 0.0003 | 1.111111111 | 1.5 | 0.0005 |
| KL02 | 0.01 | 70 | 2 | 365 | 60 | 25550 | 0.00033 | 0.0003 | 1.111111111 | 1.5 | 0.0005 |
| SG01 | 0.025 | 70 | 2 | 365 | 60 | 25550 | 0.00083 | 0.0003 | 2.777777778 | 1.5 | 0.00125 |
| SG02 | 0 | 70 | 2 | 365 | 60 | 25550 | 0.00000 | 0.0003 | 0 | 1.5 | 0 |
| KP01 | 0.01 | 70 | 2 | 365 | 60 | 25550 | 0.00033 | 0.0003 | 1.111111111 | 1.5 | 0.0005 |
| KP02 | 0 | 70 | 2 | 365 | 60 | 25550 | 0.00000 | 0.0003 | 0 | 1.5 | 0 |
| MP01 | 0.01 | 70 | 2 | 365 | 60 | 25550 | 0.00033 | 0.0003 | 1.111111111 | 1.5 | 0.0005 |
| MP02 | 0.01 | 70 | 2 | 365 | 60 | 25550 | 0.00033 | 0.0003 | 1.111111111 | 1.5 | 0.0005 |
| RJ01 | 0.005 | 70 | 2 | 365 | 60 | 25550 | 0.00017 | 0.0003 | 0.555555556 | 1.5 | 0.00025 |
| RJ02 | 0.005 | 70 | 2 | 365 | 60 | 25550 | 0.00017 | 0.0003 | 0.555555556 | 1.5 | 0.00025 |
| KB01 | 0.025 | 70 | 2 | 365 | 60 | 25550 | 0.00083 | 0.0003 | 2.777777778 | 1.5 | 0.00125 |
| KB02 | 0.025 | 70 | 2 | 365 | 60 | 25550 | 0.00083 | 0.0003 | 2.777777778 | 1.5 | 0.00125 |
| DP01 | 0 | 70 | 2 | 365 | 60 | 25550 | 0.00000 | 0.0003 | 0 | 1.5 | 0 |
| DP02 | 0.005 | 70 | 2 | 365 | 60 | 25550 | 0.00017 | 0.0003 | 0.555555556 | 1.5 | 0.00025 |
| KP01 | 0 | 70 | 2 | 365 | 60 | 25550 | 0.00000 | 0.0003 | 0 | 1.5 | 0 |
| KP02 | 0 | 70 | 2 | 365 | 60 | 25550 | 0.00000 | 0.0003 | 0 | 1.5 | 0 |
| RD01 | 0 | 70 | 2 | 365 | 60 | 25550 | 0.00000 | 0.0003 | 0 | 1.5 | 0 |
| RD02 | 0.005 | 70 | 2 | 365 | 60 | 25550 | 0.00017 | 0.0003 | 0.555555556 | 1.5 | 0.00025 |
| AM01 | 0.05 | 70 | 2 | 365 | 60 | 25550 | 0.00167 | 0.0003 | 5.555555556 | 1.5 | 0.0025 |
| NN01 | 0.025 | 70 | 2 | 365 | 60 | 25550 | 0.00083 | 0.0003 | 2.777777778 | 1.5 | 0.00125 |
| MP01 | 0.005 | 70 | 2 | 365 | 60 | 25550 | 0.00017 | 0.0003 | 0.555555556 | 1.5 | 0.00025 |
| NN02 | 0 | 70 | 2 | 365 | 60 | 25550 | 0.00000 | 0.0003 | 0 | 1.5 | 0 |
| BP01 | 0 | 70 | 2 | 365 | 60 | 25550 | 0.00000 | 0.0003 | 0 | 1.5 | 0 |
| MP02 | 0.01 | 70 | 2 | 365 | 60 | 25550 | 0.00033 | 0.0003 | 1.111111111 | 1.5 | 0.0005 |
| MJL01 | 0.005 | 70 | 2 | 365 | 60 | 25550 | 0.00017 | 0.0003 | 0.555555556 | 1.5 | 0.00025 |
| MJL02 | 0.005 | 70 | 2 | 365 | 60 | 25550 | 0.00017 | 0.0003 | 0.555555556 | 1.5 | 0.00025 |
| GPL01 | 0.025 | 70 | 2 | 365 | 60 | 25550 | 0.00083 | 0.0003 | 2.777777778 | 1.5 | 0.00125 |
| MTR01 | 0 | 70 | 2 | 365 | 60 | 25550 | 0.00000 | 0.0003 | 0 | 1.5 | 0 |
| GPL02 | 0 | 70 | 2 | 365 | 60 | 25550 | 0.00000 | 0.0003 | 0 | 1.5 | 0 |
| CCR01 | 0.01 | 70 | 2 | 365 | 60 | 25550 | 0.00033 | 0.0003 | 1.111111111 | 1.5 | 0.0005 |
| CCR02 | 0.5 | 70 | 2 | 365 | 60 | 25550 | 0.01667 | 0.0003 | 55.55555556 | 1.5 | 0.025 |
| KTP01 | 0 | 70 | 2 | 365 | 60 | 25550 | 0.00000 | 0.0003 | 0 | 1.5 | 0 |
| BJP01 | 0.005 | 70 | 2 | 365 | 60 | 25550 | 0.00017 | 0.0003 | 0.555555556 | 1.5 | 0.00025 |
| KHG01 | 0.01 | 70 | 2 | 365 | 60 | 25550 | 0.00033 | 0.0003 | 1.111111111 | 1.5 | 0.0005 |
| GBP01 | 0 | 70 | 2 | 365 | 60 | 25550 | 0.00000 | 0.0003 | 0 | 1.5 | 0 |
| JWN01 | 0 | 70 | 2 | 365 | 60 | 25550 | 0.00000 | 0.0003 | 0 | 1.5 | 0 |
| HNM01 | 0.01 | 70 | 2 | 365 | 60 | 25550 | 0.00033 | 0.0003 | 1.111111111 | 1.5 | 0.0005 |
| SP01 | 0.025 | 70 | 2 | 365 | 60 | 25550 | 0.00083 | 0.0003 | 2.777777778 | 1.5 | 0.00125 |
| CKS01 | 0.05 | 70 | 2 | 365 | 60 | 25550 | 0.00167 | 0.0003 | 5.555555556 | 1.5 | 0.0025 |
| PKL01 | 0.005 | 70 | 2 | 365 | 60 | 25550 | 0.00017 | 0.0003 | 0.555555556 | 1.5 | 0.00025 |
| PPD01 | 0.01 | 70 | 2 | 365 | 60 | 25550 | 0.00033 | 0.0003 | 1.111111111 | 1.5 | 0.0005 |
| PPD02 | 0 | 70 | 2 | 365 | 60 | 25550 | 0.00000 | 0.0003 | 0 | 1.5 | 0 |
| DLP01 | 0.025 | 70 | 2 | 365 | 60 | 25550 | 0.00083 | 0.0003 | 2.777777778 | 1.5 | 0.00125 |
| BSP01 | 0.01 | 70 | 2 | 365 | 60 | 25550 | 0.00033 | 0.0003 | 1.111111111 | 1.5 | 0.0005 |
| KTP02 | 0 | 70 | 2 | 365 | 60 | 25550 | 0.00000 | 0.0003 | 0 | 1.5 | 0 |
| BDP01 | 0.05 | 70 | 2 | 365 | 60 | 25550 | 0.00167 | 0.0003 | 5.555555556 | 1.5 | 0.0025 |
| SDP01 | 0.01 | 70 | 2 | 365 | 60 | 25550 | 0.00033 | 0.0003 | 1.111111111 | 1.5 | 0.0005 |
|  |  |  |  |  |  |  |  |  |  |  |  |
|  |  |  |  |  |  | **Average** | **0.00084** |  | **2.785947712** |  | **0.001253676** |

Table: Cumulative Average Daily Dose through oral and dermal for male, female and child

|  | **Male** | |  |  |  | **Female** | |  |  |  | **Child** | | |  |  |
| --- | --- | --- | --- | --- | --- | --- | --- | --- | --- | --- | --- | --- | --- | --- | --- |
| **Sampling Site** | **ADD Oral** | **ADD dermal** | **Total ADD** | **Mean** | **Median** | **ADD Oral** | **ADD dermal** | **Cumulative** | **Mean** | **Median** | **ADD Oral** | **ADD dermal** | **Cumulative** | **Mean** | **Median** |
| KP1 | 0.000153846 | 9.83077E-06 | 0.000163677 | 8.18385E-05 | 8.18385E-05 | 0.000166667 | 0.00001065 | 0.00017732 | 4.21307E-05 | 8.86583E-05 | 0.000106667 | 0.00000594 | 0.000112607 | 2.51714E-05 | 5.63033E-05 |
| KP2 | 0.000153846 | 9.83077E-06 | 0.000163677 | 8.18385E-05 | 8.18385E-05 | 0.000166667 | 0.00001065 | 0.00017732 | 4.21307E-05 | 8.86583E-05 | 0.000106667 | 0.00000594 | 0.000112607 | 2.51714E-05 | 5.63033E-05 |
| KP3 | 0.001538462 | 9.83077E-05 | 0.001636769 | 0.000818385 | 0.000818385 | 0.001666667 | 0.0001065 | 0.00177317 | 0.000421307 | 0.000886583 | 0.001066667 | 0.0000594 | 0.001126067 | 0.000251714 | 0.000563033 |
| RP1 | 0.000307692 | 1.96615E-05 | 0.000327354 | 0.000163677 | 0.000163677 | 0.000333333 | 0.0000213 | 0.00035463 | 8.42615E-05 | 0.000177317 | 0.000213333 | 0.00001188 | 0.000225213 | 5.03428E-05 | 0.000112607 |
| RP2 | 0.000153846 | 9.83077E-06 | 0.000163677 | 8.18385E-05 | 8.18385E-05 | 0.000166667 | 0.00001065 | 0.00017732 | 4.21307E-05 | 8.86583E-05 | 0.000106667 | 0.00000594 | 0.000112607 | 2.51714E-05 | 5.63033E-05 |
| RP3 | 0.000307692 | 1.96615E-05 | 0.000327354 | 0.000163677 | 0.000163677 | 0.000333333 | 0.0000213 | 0.00035463 | 8.42615E-05 | 0.000177317 | 0.000213333 | 0.00001188 | 0.000225213 | 5.03428E-05 | 0.000112607 |
| PK01 | 0.000769231 | 4.91538E-05 | 0.000818385 | 0.000409192 | 0.000409192 | 0.000833333 | 0.00005325 | 0.00088658 | 0.000210654 | 0.000443292 | 0.000533333 | 0.0000297 | 0.000563033 | 0.000125857 | 0.000281517 |
| PK02 | 0.000769231 | 4.91538E-05 | 0.000818385 | 0.000409192 | 0.000409192 | 0.000833333 | 0.00005325 | 0.00088658 | 0.000210654 | 0.000443292 | 0.000533333 | 0.0000297 | 0.000563033 | 0.000125857 | 0.000281517 |
| PK03 | 0 | 0 | 0 | 0 | 0 | 0 | 0 | 0 | 0 | 0 | 0 | 0 | 0 | 0 | 0 |
| PK04 | 0.000307692 | 1.96615E-05 | 0.000327354 | 0.000163677 | 0.000163677 | 0.000333333 | 0.0000213 | 0.00035463 | 8.42615E-05 | 0.000177317 | 0.000213333 | 0.00001188 | 0.000225213 | 5.03428E-05 | 0.000112607 |
| BP01 | 0.000153846 | 9.83077E-06 | 0.000163677 | 8.18385E-05 | 8.18385E-05 | 0.000166667 | 0.00001065 | 0.00017732 | 4.21307E-05 | 8.86583E-05 | 0.000106667 | 0.00000594 | 0.000112607 | 2.51714E-05 | 5.63033E-05 |
| BP02 | 0.000153846 | 9.83077E-06 | 0.000163677 | 8.18385E-05 | 8.18385E-05 | 0.000166667 | 0.00001065 | 0.00017732 | 4.21307E-05 | 8.86583E-05 | 0.000106667 | 0.00000594 | 0.000112607 | 2.51714E-05 | 5.63033E-05 |
| BP03 | 0.000307692 | 1.96615E-05 | 0.000327354 | 0.000163677 | 0.000163677 | 0.000333333 | 0.0000213 | 0.00035463 | 8.42615E-05 | 0.000177317 | 0.000213333 | 0.00001188 | 0.000225213 | 5.03428E-05 | 0.000112607 |
| BP04 | 0 | 0 | 0 | 0 | 0 | 0 | 0 | 0 | 0 | 0 | 0 | 0 | 0 | 0 | 0 |
| BP05 | 0.000153846 | 9.83077E-06 | 0.000163677 | 8.18385E-05 | 8.18385E-05 | 0.000166667 | 0.00001065 | 0.00017732 | 4.21307E-05 | 8.86583E-05 | 0.000106667 | 0.00000594 | 0.000112607 | 2.51714E-05 | 5.63033E-05 |
| HN01 | 0.015384615 | 0.000983077 | 0.016367692 | 0.008183846 | 0.008183846 | 0.016666667 | 0.001065 | 0.01773167 | 0.004213075 | 0.008865833 | 0.010666667 | 0.000594 | 0.011260667 | 0.002517141 | 0.005630333 |
| ML01 | 0.000153846 | 9.83077E-06 | 0.000163677 | 8.18385E-05 | 8.18385E-05 | 0.000166667 | 0.00001065 | 0.00017732 | 4.21307E-05 | 8.86583E-05 | 0.000106667 | 0.00000594 | 0.000112607 | 2.51714E-05 | 5.63033E-05 |
| ML02 | 0.000153846 | 9.83077E-06 | 0.000163677 | 8.18385E-05 | 8.18385E-05 | 0.000166667 | 0.00001065 | 0.00017732 | 4.21307E-05 | 8.86583E-05 | 0.000106667 | 0.00000594 | 0.000112607 | 2.51714E-05 | 5.63033E-05 |
| ML03 | 0.000769231 | 4.91538E-05 | 0.000818385 | 0.000409192 | 0.000409192 | 0.000833333 | 0.00005325 | 0.00088658 | 0.000210654 | 0.000443292 | 0.000533333 | 0.0000297 | 0.000563033 | 0.000125857 | 0.000281517 |
| DN01 | 0.000153846 | 9.83077E-06 | 0.000163677 | 8.18385E-05 | 8.18385E-05 | 0.000166667 | 0.00001065 | 0.00017732 | 4.21307E-05 | 8.86583E-05 | 0.000106667 | 0.00000594 | 0.000112607 | 2.51714E-05 | 5.63033E-05 |
| DN02 | 0.000153846 | 9.83077E-06 | 0.000163677 | 8.18385E-05 | 8.18385E-05 | 0.000166667 | 0.00001065 | 0.00017732 | 4.21307E-05 | 8.86583E-05 | 0.000106667 | 0.00000594 | 0.000112607 | 2.51714E-05 | 5.63033E-05 |
| KL01 | 0.000307692 | 1.96615E-05 | 0.000327354 | 0.000163677 | 0.000163677 | 0.000333333 | 0.0000213 | 0.00035463 | 8.42615E-05 | 0.000177317 | 0.000213333 | 0.00001188 | 0.000225213 | 5.03428E-05 | 0.000112607 |
| KL02 | 0.000307692 | 1.96615E-05 | 0.000327354 | 0.000163677 | 0.000163677 | 0.000333333 | 0.0000213 | 0.00035463 | 8.42615E-05 | 0.000177317 | 0.000213333 | 0.00001188 | 0.000225213 | 5.03428E-05 | 0.000112607 |
| SG01 | 0.000769231 | 4.91538E-05 | 0.000818385 | 0.000409192 | 0.000409192 | 0.000833333 | 0.00005325 | 0.00088658 | 0.000210654 | 0.000443292 | 0.000533333 | 0.0000297 | 0.000563033 | 0.000125857 | 0.000281517 |
| SG02 | 0 | 0 | 0 | 0 | 0 | 0 | 0 | 0 | 0 | 0 | 0 | 0 | 0 | 0 | 0 |
| KP01 | 0.000307692 | 1.96615E-05 | 0.000327354 | 0.000163677 | 0.000163677 | 0.000333333 | 0.0000213 | 0.00035463 | 8.42615E-05 | 0.000177317 | 0.000213333 | 0.00001188 | 0.000225213 | 5.03428E-05 | 0.000112607 |
| KP02 | 0 | 0 | 0 | 0 | 0 | 0 | 0 | 0 | 0 | 0 | 0 | 0 | 0 | 0 | 0 |
| MP01 | 0.000307692 | 1.96615E-05 | 0.000327354 | 0.000163677 | 0.000163677 | 0.000333333 | 0.0000213 | 0.00035463 | 8.42615E-05 | 0.000177317 | 0.000213333 | 0.00001188 | 0.000225213 | 5.03428E-05 | 0.000112607 |
| MP02 | 0.000307692 | 1.96615E-05 | 0.000327354 | 0.000163677 | 0.000163677 | 0.000333333 | 0.0000213 | 0.00035463 | 8.42615E-05 | 0.000177317 | 0.000213333 | 0.00001188 | 0.000225213 | 5.03428E-05 | 0.000112607 |
| RJ01 | 0.000153846 | 9.83077E-06 | 0.000163677 | 8.18385E-05 | 8.18385E-05 | 0.000166667 | 0.00001065 | 0.00017732 | 4.21307E-05 | 8.86583E-05 | 0.000106667 | 0.00000594 | 0.000112607 | 2.51714E-05 | 5.63033E-05 |
| RJ02 | 0.000153846 | 9.83077E-06 | 0.000163677 | 8.18385E-05 | 8.18385E-05 | 0.000166667 | 0.00001065 | 0.00017732 | 4.21307E-05 | 8.86583E-05 | 0.000106667 | 0.00000594 | 0.000112607 | 2.51714E-05 | 5.63033E-05 |
| KB01 | 0.000769231 | 4.91538E-05 | 0.000818385 | 0.000409192 | 0.000409192 | 0.000833333 | 0.00005325 | 0.00088658 | 0.000210654 | 0.000443292 | 0.000533333 | 0.0000297 | 0.000563033 | 0.000125857 | 0.000281517 |
| KB02 | 0.000769231 | 4.91538E-05 | 0.000818385 | 0.000409192 | 0.000409192 | 0.000833333 | 0.00005325 | 0.00088658 | 0.000210654 | 0.000443292 | 0.000533333 | 0.0000297 | 0.000563033 | 0.000125857 | 0.000281517 |
| DP01 | 0 | 0 | 0 | 0 | 0 | 0 | 0 | 0 | 0 | 0 | 0 | 0 | 0 | 0 | 0 |
| DP02 | 0.000153846 | 9.83077E-06 | 0.000163677 | 8.18385E-05 | 8.18385E-05 | 0.000166667 | 0.00001065 | 0.00017732 | 4.21307E-05 | 8.86583E-05 | 0.000106667 | 0.00000594 | 0.000112607 | 2.51714E-05 | 5.63033E-05 |
| KP01 | 0 | 0 | 0 | 0 | 0 | 0 | 0 | 0 | 0 | 0 | 0 | 0 | 0 | 0 | 0 |
| KP02 | 0 | 0 | 0 | 0 | 0 | 0 | 0 | 0 | 0 | 0 | 0 | 0 | 0 | 0 | 0 |
| RD01 | 0 | 0 | 0 | 0 | 0 | 0 | 0 | 0 | 0 | 0 | 0 | 0 | 0 | 0 | 0 |
| RD02 | 0.000153846 | 9.83077E-06 | 0.000163677 | 8.18385E-05 | 8.18385E-05 | 0.000166667 | 0.00001065 | 0.00017732 | 4.21307E-05 | 8.86583E-05 | 0.000106667 | 0.00000594 | 0.000112607 | 2.51714E-05 | 5.63033E-05 |
| AM01 | 0.001538462 | 9.83077E-05 | 0.001636769 | 0.000818385 | 0.000818385 | 0.001666667 | 0.0001065 | 0.00177317 | 0.000421307 | 0.000886583 | 0.001066667 | 0.0000594 | 0.001126067 | 0.000251714 | 0.000563033 |
| NN01 | 0.000769231 | 4.91538E-05 | 0.000818385 | 0.000409192 | 0.000409192 | 0.000833333 | 0.00005325 | 0.00088658 | 0.000210654 | 0.000443292 | 0.000533333 | 0.0000297 | 0.000563033 | 0.000125857 | 0.000281517 |
| MP01 | 0.000153846 | 9.83077E-06 | 0.000163677 | 8.18385E-05 | 8.18385E-05 | 0.000166667 | 0.00001065 | 0.00017732 | 4.21307E-05 | 8.86583E-05 | 0.000106667 | 0.00000594 | 0.000112607 | 2.51714E-05 | 5.63033E-05 |
| NN02 | 0 | 0 | 0 | 0 | 0 | 0 | 0 | 0 | 0 | 0 | 0 | 0 | 0 | 0 | 0 |
| BP01 | 0 | 0 | 0 | 0 | 0 | 0 | 0 | 0 | 0 | 0 | 0 | 0 | 0 | 0 | 0 |
| MP02 | 0.000307692 | 1.96615E-05 | 0.000327354 | 0.000163677 | 0.000163677 | 0.000333333 | 0.0000213 | 0.00035463 | 8.42615E-05 | 0.000177317 | 0.000213333 | 0.00001188 | 0.000225213 | 5.03428E-05 | 0.000112607 |
| MJL01 | 0.000153846 | 9.83077E-06 | 0.000163677 | 8.18385E-05 | 8.18385E-05 | 0.000166667 | 0.00001065 | 0.00017732 | 4.21307E-05 | 8.86583E-05 | 0.000106667 | 0.00000594 | 0.000112607 | 2.51714E-05 | 5.63033E-05 |
| MJL02 | 0.000153846 | 9.83077E-06 | 0.000163677 | 8.18385E-05 | 8.18385E-05 | 0.000166667 | 0.00001065 | 0.00017732 | 4.21307E-05 | 8.86583E-05 | 0.000106667 | 0.00000594 | 0.000112607 | 2.51714E-05 | 5.63033E-05 |
| GPL01 | 0.000769231 | 4.91538E-05 | 0.000818385 | 0.000409192 | 0.000409192 | 0.000833333 | 0.00005325 | 0.00088658 | 0.000210654 | 0.000443292 | 0.000533333 | 0.0000297 | 0.000563033 | 0.000125857 | 0.000281517 |
| MTR01 | 0 | 0 | 0 | 0 | 0 | 0 | 0 | 0 | 0 | 0 | 0 | 0 | 0 | 0 | 0 |
| GPL02 | 0 | 0 | 0 | 0 | 0 | 0 | 0 | 0 | 0 | 0 | 0 | 0 | 0 | 0 | 0 |
| CCR01 | 0.000307692 | 1.96615E-05 | 0.000327354 | 0.000163677 | 0.000163677 | 0.000333333 | 0.0000213 | 0.00035463 | 8.42615E-05 | 0.000177317 | 0.000213333 | 0.00001188 | 0.000225213 | 5.03428E-05 | 0.000112607 |
| CCR02 | 0.015384615 | 0.000983077 | 0.016367692 | 0.008183846 | 0.008183846 | 0.016666667 | 0.001065 | 0.01773167 | 0.004213075 | 0.008865833 | 0.010666667 | 0.000594 | 0.011260667 | 0.002517141 | 0.005630333 |
| KTP01 | 0 | 0 | 0 | 0 | 0 | 0 | 0 | 0 | 0 | 0 | 0 | 0 | 0 | 0 | 0 |
| BJP01 | 0.000153846 | 9.83077E-06 | 0.000163677 | 8.18385E-05 | 8.18385E-05 | 0.000166667 | 0.00001065 | 0.00017732 | 4.21307E-05 | 8.86583E-05 | 0.000106667 | 0.00000594 | 0.000112607 | 2.51714E-05 | 5.63033E-05 |
| KHG01 | 0.000307692 | 1.96615E-05 | 0.000327354 | 0.000163677 | 0.000163677 | 0.000333333 | 0.0000213 | 0.00035463 | 8.42615E-05 | 0.000177317 | 0.000213333 | 0.00001188 | 0.000225213 | 5.03428E-05 | 0.000112607 |
| GBP01 | 0 | 0 | 0 | 0 | 0 | 0 | 0 | 0 | 0 | 0 | 0 | 0 | 0 | 0 | 0 |
| JWN01 | 0 | 0 | 0 | 0 | 0 | 0 | 0 | 0 | 0 | 0 | 0 | 0 | 0 | 0 | 0 |
| HNM01 | 0.000307692 | 1.96615E-05 | 0.000327354 | 0.000163677 | 0.000163677 | 0.000333333 | 0.0000213 | 0.00035463 | 8.42615E-05 | 0.000177317 | 0.000213333 | 0.00001188 | 0.000225213 | 5.03428E-05 | 0.000112607 |
| SP01 | 0.000769231 | 4.91538E-05 | 0.000818385 | 0.000409192 | 0.000409192 | 0.000833333 | 0.00005325 | 0.00088658 | 0.000210654 | 0.000443292 | 0.000533333 | 0.0000297 | 0.000563033 | 0.000125857 | 0.000281517 |
| CKS01 | 0.001538462 | 9.83077E-05 | 0.001636769 | 0.000818385 | 0.000818385 | 0.001666667 | 0.0001065 | 0.00177317 | 0.000421307 | 0.000886583 | 0.001066667 | 0.0000594 | 0.001126067 | 0.000251714 | 0.000563033 |
| PKL01 | 0.000153846 | 9.83077E-06 | 0.000163677 | 8.18385E-05 | 8.18385E-05 | 0.000166667 | 0.00001065 | 0.00017732 | 4.21307E-05 | 8.86583E-05 | 0.000106667 | 0.00000594 | 0.000112607 | 2.51714E-05 | 5.63033E-05 |
| PPD01 | 0.000307692 | 1.96615E-05 | 0.000327354 | 0.000163677 | 0.000163677 | 0.000333333 | 0.0000213 | 0.00035463 | 8.42615E-05 | 0.000177317 | 0.000213333 | 0.00001188 | 0.000225213 | 5.03428E-05 | 0.000112607 |
| PPD02 | 0 | 0 | 0 | 0 | 0 | 0 | 0 | 0 | 0 | 0 | 0 | 0 | 0 | 0 | 0 |
| DLP01 | 0.000769231 | 4.91538E-05 | 0.000818385 | 0.000409192 | 0.000409192 | 0.000833333 | 0.00005325 | 0.00088658 | 0.000210654 | 0.000443292 | 0.000533333 | 0.0000297 | 0.000563033 | 0.000125857 | 0.000281517 |
| BSP01 | 0.000307692 | 1.96615E-05 | 0.000327354 | 0.000163677 | 0.000163677 | 0.000333333 | 0.0000213 | 0.00035463 | 8.42615E-05 | 0.000177317 | 0.000213333 | 0.00001188 | 0.000225213 | 5.03428E-05 | 0.000112607 |
| KTP02 | 0 | 0 | 0 | 0 | 0 | 0 | 0 | 0 | 0 | 0 | 0 | 0 | 0 | 0 | 0 |
| BDP01 | 0.001538462 | 9.83077E-05 | 0.001636769 | 0.000818385 | 0.000818385 | 0.001666667 | 0.0001065 | 0.00177317 | 0.000421307 | 0.000886583 | 0.001066667 | 0.0000594 | 0.001126067 | 0.000251714 | 0.000563033 |
| SDP01 | 0.000307692 | 1.96615E-05 | 0.000327354 | 0.000163677 | 0.000163677 | 0.000333333 | 0.0000213 | 0.00035463 | 8.42615E-05 | 0.000177317 | 0.000213333 | 0.00001188 | 0.000225213 | 5.03428E-05 | 0.000112607 |
|  |  |  | **Average** | **0.000410396** | **0.000410396** |  |  |  | **0.000211273** | **0.000444595** |  |  |  | **0.000126227** | **0.000282345** |
